# Lag effects of meteorological factors on the risk of daily outpatient visits for herpangina in Guangzhou (2014–2023)

**DOI:** 10.3389/fped.2026.1868116

**Published:** 2026-08-04

**Authors:** Feifei Yan, Rong Xu, Yi Dong, Yuqing Li, Xiaohan Yang, Hui Li, Minyi Situ, Kaicai Wang, Wanting Yang, Wangkai Liu

**Affiliations:** 1 Department of Pediatrics, Guangdong Women and Children Hospital, Guangzhou, China; 2 Department of Public Health, School of Medicine, Jinan University, Guangzhou, China; 3 Medical Genetic Center, Guangdong Women and Children Hospital, Southern University of Science and Technology, Guangzhou, China; 4 Department of Information Management, Guangdong Women and Children Hospital, Guangzhou, China

**Keywords:** children, distributed lag nonlinear model, Guangzhou, herpangina, meteorological factors, relative humidity, temperature, time series study

## Abstract

**Objective:**

Herpangina (HA) is a common acute infectious disease in children that may exhibit epidemiological patterns associated with meteorological factors. This study investigated the short-term and lagged associations of temperature and relative humidity with daily outpatient visits for HA in Guangzhou and explored patterns across sex and age groups.

**Methods:**

Daily outpatient visits for HA, daily mean temperature, relative humidity, and particulate matter with an aerodynamic diameter of 2.5 micrometers or less (PM_2.5_) were collected from January 1, 2014 to December 31, 2023. A quasi-Poisson generalized linear model combined with distributed lag nonlinear models was used to estimate exposure-lag-response associations. Temperature, relative humidity, and PM_2.5_ cross-basis terms were included simultaneously, with adjustment for long-term trends, seasonality, day of week, public holidays, and the COVID-19 period.

**Results:**

During the study period, 83,146 HA outpatient visits were recorded, 56.4% of which were among male patients. Daily outpatient visits showed clear seasonal variation, with larger peaks generally occurring during summer and autumn. Compared with the median temperature (24.38 °C), the cumulative RR was 0.482 (95% CI: 0.353–0.657) at the 2.5th percentile (10.77 °C) and 1.966 (95% CI: 1.714–2.254) at the 97.5th percentile (30.63 °C) over lags 0–14 days. Compared with the median relative humidity (79.15%), the cumulative RR was 0.878 (95% CI: 0.795–0.970) at the 10th percentile (62.21%) and 1.477 (95% CI: 1.297–1.682) at the 97.5th percentile (93.21%) over lags 0–20 days. High-temperature and high-humidity associations were observed across sex and age strata. The lower-humidity association was less robust in sensitivity analyses.

**Conclusion:**

High temperature and high relative humidity were associated with increased daily outpatient visits for HA in Guangzhou, whereas low temperature and lower relative humidity were associated with fewer visits in the main model. The lower-humidity estimate was less stable across alternative temporal-control settings. These findings support incorporating lagged meteorological information into HA surveillance and preparedness.

## 1 Introduction

Herpangina (HA) is an acute upper respiratory tract infection primarily caused by Coxsackievirus A group (CV-A), commonly affects children, and is characterized by fever, sore throat, and the appearance of blisters or ulcers in the posterior oral cavity, including the soft palate, uvula, and tonsils (1). The disease is contagious and mainly spreads through the fecal-oral route, respiratory droplets, or contact (2). Although HA is usually self-limiting with a good prognosis, some children may experience dehydration, febrile seizures, and, in rare cases, complications such as encephalitis or myocarditis (3). In China, HA is a common reason for pediatric outpatient visits, especially during the summer and autumn seasons when there is a significant peak in cases.

Meteorological factors play a pivotal role in the emergence and spread of various infectious diseases (4). Environmental conditions such as temperature and humidity can influence disease transmission intensity and temporal patterns through multiple pathways, including effects on viral survival and stability, human contact patterns, and host immunity (5). Previous domestic studies have indicated correlations between HA outbreaks and meteorological factors (6), yet evidence on the associations between meteorological factors and daily outpatient visits for HA, particularly their lagged associations, remains relatively limited.

Guangzhou is located in the southern coastal region of China, with a subtropical monsoon climate, evergreen throughout the year, long summers and short winters, pleasant spring and autumn seasons, and an overall warm and humid climate with concurrent rainfall and heat (7). As a densely populated metropolis, the burden of childhood infectious diseases is relatively heavy. A better understanding of local meteorological factors, especially the dynamic associations of temperature and humidity with daily outpatient visits for HA, is of great significance for establishing an early warning system based on meteorological conditions and optimizing HA prevention and control strategies. This study used daily outpatient visit data for HA and meteorological data from Guangzhou from 2014 to 2023, employing a DLNM to quantitatively analyze the exposure-lag-response associations of temperature and relative humidity with daily outpatient visits for HA and to explore patterns across sex and age groups.

## 2 Data and methods

### 2.1 Study area

Guangzhou is the capital of Guangdong Province, an important megacity and national central city in southern China (8, 9). Geographically located at the northern edge of the Pearl River Delta (north latitude 22° 26′ to 23° 56′, east longitude 112° 57′ to 114° 03′), it has long, hot, and rainy summers and short, mild, and dry winters. The study was conducted in Guangzhou, whereas the visit data were obtained from Guangdong Women and Children Hospital.

### 2.2 Data sources

#### 2.2.1 HA visit data

Daily outpatient visits for HA were extracted from the Hospital Information System (HIS) of Guangdong Women and Children Hospital from January 1, 2014 to December 31, 2023. HA outpatient visits were identified in the HIS using the International Classification of Diseases, 10th Revision (ICD-10) code B08.5 (10), which corresponds to enteroviral vesicular pharyngitis (HA). The diagnostic criteria followed the Chinese pediatric guidelines, including the 7th to 10th editions of the Pediatrics textbook and the 2019 HA Diagnosis and Treatment Guidelines (11–14). The extracted variables included visit date, sex, and age in months. The daily outcome was defined as the total number of outpatient visits for HA on each calendar day.

#### 2.2.2 Meteorological and air-pollution data

Daily mean temperature (°C), relative humidity (%), and PM_2.5_ concentration (μg/m^3^) data were obtained from the China Meteorological Data Network (http://data.cma.cn/). PM_2.5_ was the only air-pollution variable included in the analysis, because fine particulate matter may be associated with respiratory infection-related healthcare outpatient visits and may also vary with meteorological conditions. The meteorological and PM_2.5_ data were complete during the study period, with no missing daily values.

### 2.3 Statistical methods

#### 2.3.1 Descriptive and correlation analysis

We summarized daily outpatient visits for HA, meteorological variables, and PM_2.5_ concentrations using means, standard deviations, minimums, maximums, percentiles, and interquartile ranges. Spearman correlation coefficients were calculated to describe pairwise correlations among daily HA outpatient visits, temperature, relative humidity, and PM_2.5_. Pairwise correlations were also examined to assess potential multicollinearity among the exposure and covariate variables, with an absolute correlation coefficient below 0.75 considered acceptable.

#### 2.3.2 Distributed lag nonlinear model (DLNM)

Given the overdispersion of the daily counts of outpatient visits for HA and the potentially nonlinear and delayed associations with meteorological exposures, we fitted a generalized linear model with a quasi-Poisson distribution and log link, combined with distributed lag nonlinear models (DLNMs). Temperature, relative humidity, and PM_2.5_ were represented by cross-basis functions and entered simultaneously into the same model. The core model formula is as follows:

$$\begin{array}{rl} & Y_{t}∼quasi\;Poisson(\mu_{t})\\ & \log\;(\mu_{t})=\alpha +CB_{Temp}(Temp_{t};\;14,3,3)\\ & +CB_{RH}(RH_{t};\;20,2,3)+CB_{PM2.5}(PM_{2.5,t};\;15,3,4)\\ & +s_{cyclic}(DOY_{t},k=8)+ns(Time_{t},df=100)\\ & +\overset{7}{\underset{j=2}{\sum }}\;\beta_{j}DOW_{jt}+\beta_{H}Holiday_{t}+\beta_{C}COVID_{t} \end{array}$$

Here, $Y_{t}$ denotes the number of outpatient visits for HA on day t, $\;CB_{Temp}$, $CB_{RH}$, and $CB_{PM2.5}$ denote the cross-basis functions for daily mean temperature, relative humidity, and PM_2.5_, respectively; $ns(Time_{t},df=100)$ denotes a natural cubic spline of calendar time with 10 degrees of freedom per year over the 10-year study period; $s_{cyclic}(DOY_{t},k=8)$ denotes a cyclic cubic regression spline for day of year to control seasonal patterns; $DOW_{jt}$ denotes day of week; $Holiday_{t}$ denotes public holidays; and $COVID_{t}$ denotes the COVID-19 control period.

Natural cubic splines were used for both the exposure-response and lag-response dimensions of each cross-basis. For temperature, the exposure-response and lag-response dimensions were specified with 3° of freedom each, and the maximum lag was set to 14 days. For relative humidity, the exposure-response and lag-response dimensions were specified with 2 and 3° of freedom, respectively, and the maximum lag was set to 20 days. PM_2.5_ was included as a potential confounder using 3° of freedom for the concentration-response dimension and 4 degrees of freedom for the lag-response dimension over lags 0–15 days. Candidate maximum lag windows and spline degrees of freedom were assessed before specifying the primary model. Model selection was based on Q-AIC, prior evidence regarding plausible lag structures, model parsimony, and the stability and interpretability of cumulative estimates, rather than on the minimum Q-AIC alone. Exposure-specific lag windows were selected because temperature, relative humidity, and PM_2.5_ may operate over different temporal scales. For temperature, although longer lag windows slightly reduced Q-AIC, they produced less precise cumulative estimates and greater uncertainty at longer lags. A 14-day window was therefore selected to capture the main delayed association while maintaining model stability and parsimony. For relative humidity, the cumulative high-humidity estimate stabilized at approximately lags 18–20; extending the lag beyond 20 days did not improve Q-AIC and increased uncertainty. PM_2.5_ was included as a potential confounder rather than a primary exposure, and its lag window was fixed at 0–15 days based on prior air-pollution studies and model parsimony. Further details are provided in Supplementary Table S1.

The COVID-19 control period was defined as January 30, 2020 to May 5, 2023 (15) a period characterized by major public-health interventions in Guangzhou, during which healthcare-seeking behavior, social contact patterns, and the circulation of common pediatric infections may have differed substantially from those in other periods. Temperature associations were centered at the median temperature of 24.38 °C and were reported at the 2.5th percentile (10.77 °C) and the 97.5th percentile (30.63 °C). Relative-humidity associations were centered at the median relative humidity of 79.15% and were reported at the 10th percentile (62.21%) and the 97.5th percentile (93.21%). Stratified analyses were conducted by sex and age group (<36 and ≥36 months). Separate quasi-Poisson DLNMs were fitted for each stratum using the same cross-basis specifications, lag windows, temporal controls, and covariates as in the primary model. All analyses were conducted using R version 4.4.3.

### 2.4 Sensitivity analysis

Several sensitivity analyses were conducted to assess the robustness of the primary results. We varied long-term temporal control from 6 to 10 degrees of freedom per year and the cyclic seasonal spline across *k* = 6, 8, and 10, generating 15 alternative temporal-control specifications. To assess mutual confounding between temperature and relative humidity, we fitted separate-exposure models by omitting one meteorological cross-basis at a time while retaining the PM_2.5_ cross-basis and all temporal and calendar covariates. We also refitted the DLNM after excluding the COVID-19 control period, treating the pre-COVID and post-COVID observations as independent time-series segments to avoid carrying lagged exposures across the three-year gap. The same exposure contrasts and reference values as in the primary analysis were retained. Full results are provided in Supplementary Table S2.

## 3 Results

### 3.1 Descriptive statistics

From 2014 to 2023, 83,146 outpatient visits for HA were recorded at Guangdong Women and Children Hospital. Males accounted for 56.4% of visits. Daily outpatient visits ranged from 0 to 225, with a mean of 22.77 visits per day. Children aged less than 36 months accounted for 55.1% of visits, and children aged 36 months or older accounted for 44.9%. The mean daily temperature was 23.12 °C, the mean relative humidity was 77.30%, and daily PM_2.5_ concentrations ranged from 2.77 to 132.60 μg/m^3^ (Table 1). The maximum PM_2.5_ concentration occurred on January 6, 2014 and was accompanied by several elevated values on adjacent days, indicating that it was not an isolated value. Mean daily outpatient visits were 27.2 before the COVID-19 control period, decreased to 11.3 during January 30, 2020 to May 5, 2023, and returned to 29.9 in 2023.

**Table 1:** Descriptive statistics of daily outpatient visits for HA, meteorological variables, and PM_2.5_ concentrations in Guangzhou, 2014–2023.

Variables	Number (%)	Mean ± SD	Centiles					
			Min	25%	50%	75%	Max	IQR
Meteorological variables								
Temperature (°C)	/	23.12 ± 5.79	4.05	18.83	24.38	28.1	32.35	9.27
Relative humidity (%)	/	77.30 ± 11.07	26.93	72.09	79.15	85.09	97.09	13.0
Pollutant concentration								
PM_2.5_ (μg/m^3^)	/	29.66 ± 16.18	2.77	17.72	26.3	38.03	132.6	20.31
Outpatient visits distribution								
HA visits	83,146 (100.0)	22.77 ± 32.40	0	5	10	23	225	18
Male	46,889 (56.4)	12.84 ± 18.49	0	2	6	13	121	11
Female	36,257 (43.6)	9.93 ± 14.26	0	2	5	10	111	8
≥36 months	37,333 (44.9)	10.22 ± 17.94	0	2	4	9	170	7
<36 months	45,813 (55.1)	12.54 ± 16.87	0	2	6	14	130	12

PM_2.5_, particles with an aerodynamic diameter less than 2.5 μm; HA, herpangina; Min, minimum; Max, maximum; IQR, interquartile range.

### 3.2 Time series

**Figure 1:**
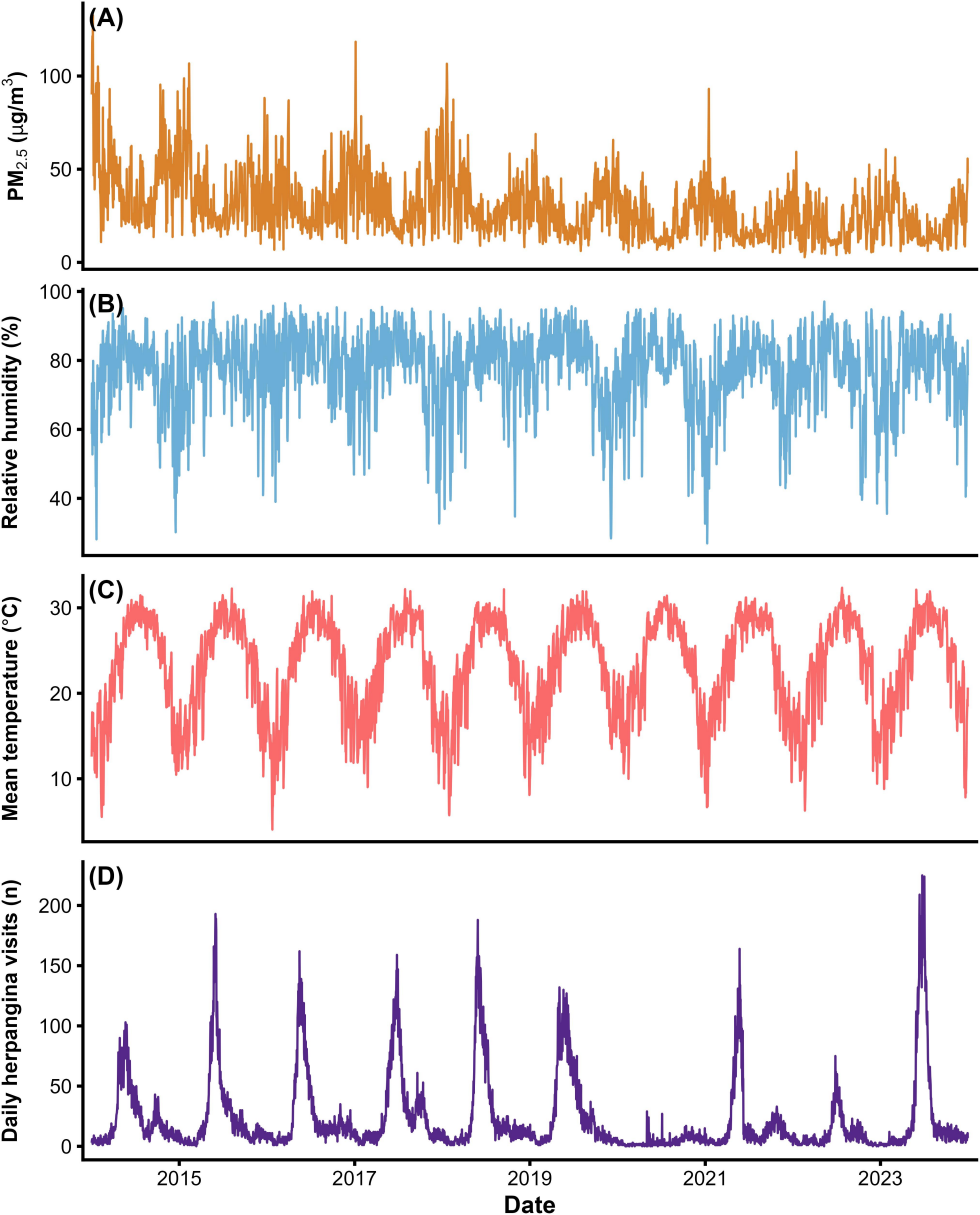
Daily time series of **(A)** PM2.5 concentration, **(B)** relative humidity, **(C)** mean temperature, and **(D)** outpatient visits for HA in Guangzhou from January 1, 2014 to December 31, 2023.

Figure 1 presents the time series of daily outpatient visits for HA, temperature, relative humidity, and PM_2.5_ in Guangzhou from 2014 to 2023. Daily outpatient visits showed clear seasonal variation, with larger peaks generally occurring during summer and autumn. PM_2.5_ displayed a different seasonal pattern, with relatively higher concentrations during winter and spring (Figure 1).

### 3.3 Correlations among meteorological factors, PM_2.5_, and daily outpatient visits for HA

Table 2 presents the Spearman correlation coefficients among daily outpatient visits for HA, temperature, relative humidity, and PM_2.5_. Daily outpatient visits were positively correlated with temperature (*r* = 0.552, *P* < 0.001) and relative humidity (*r* = 0.195, *P* < 0.001), and weakly negatively correlated with PM_2.5_ (*r* = −0.141, *P* < 0.001). Among the environmental variables, temperature was positively correlated with relative humidity (*r* = 0.280, *P* < 0.001), whereas PM_2.5_ was negatively correlated with temperature (*r* = −0.302, *P* < 0.001) and relative humidity (*r* = −0.369, *P* < 0.001). All absolute pairwise correlations among the environmental variables were below 0.75, providing no indication of strong pairwise collinearity.

**Table 2:** Spearman correlation coefficients among daily outpatient visits for HA, meteorological variables, and PM_2.5_ concentrations in Guangzhou, 2014–2023.

Variable	Temperature (°C)	Relative humidity (%)	PM_2.5_ (μg/m^3^)	HA outpatient visits
Temperature (°C)	1.000			
Relative humidity (%)	0.280*	1.000		
PM_2.5_ (μg/m^3^)	−0.302*	−0.369*	1.000	
HA outpatient visits	0.552*	0.195*	−0.141*	1.000

* *P* < 0.001.

### 3.4 Associations of meteorological factors with daily outpatient visits for HA

**Figure 2:**
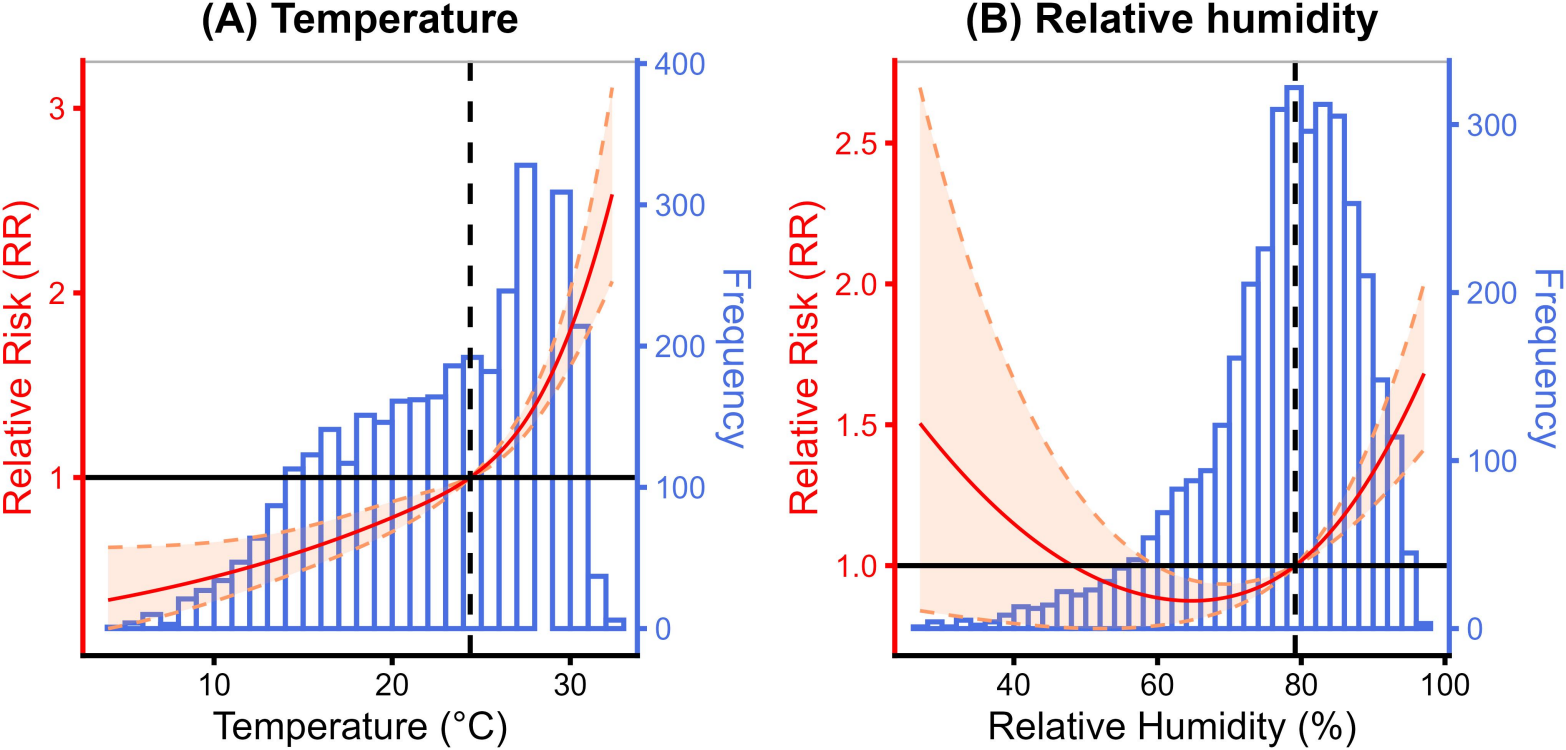
Cumulative exposure-response associations of **(A)** temperature and **(B)** relative humidity with daily outpatient visits for HA.

Figure 2 shows the cumulative exposure-response associations of temperature and relative humidity with daily outpatient visits for HA. Using the median temperature of 24.38 °C as the reference, the RR was below 1 at lower temperatures and increased above 1 at higher temperatures. Using the median relative humidity of 79.15% as the reference, the curve was below 1 across much of the lower-humidity range and increased above 1 at higher humidity levels (Figure 2).

**Figure 3:**
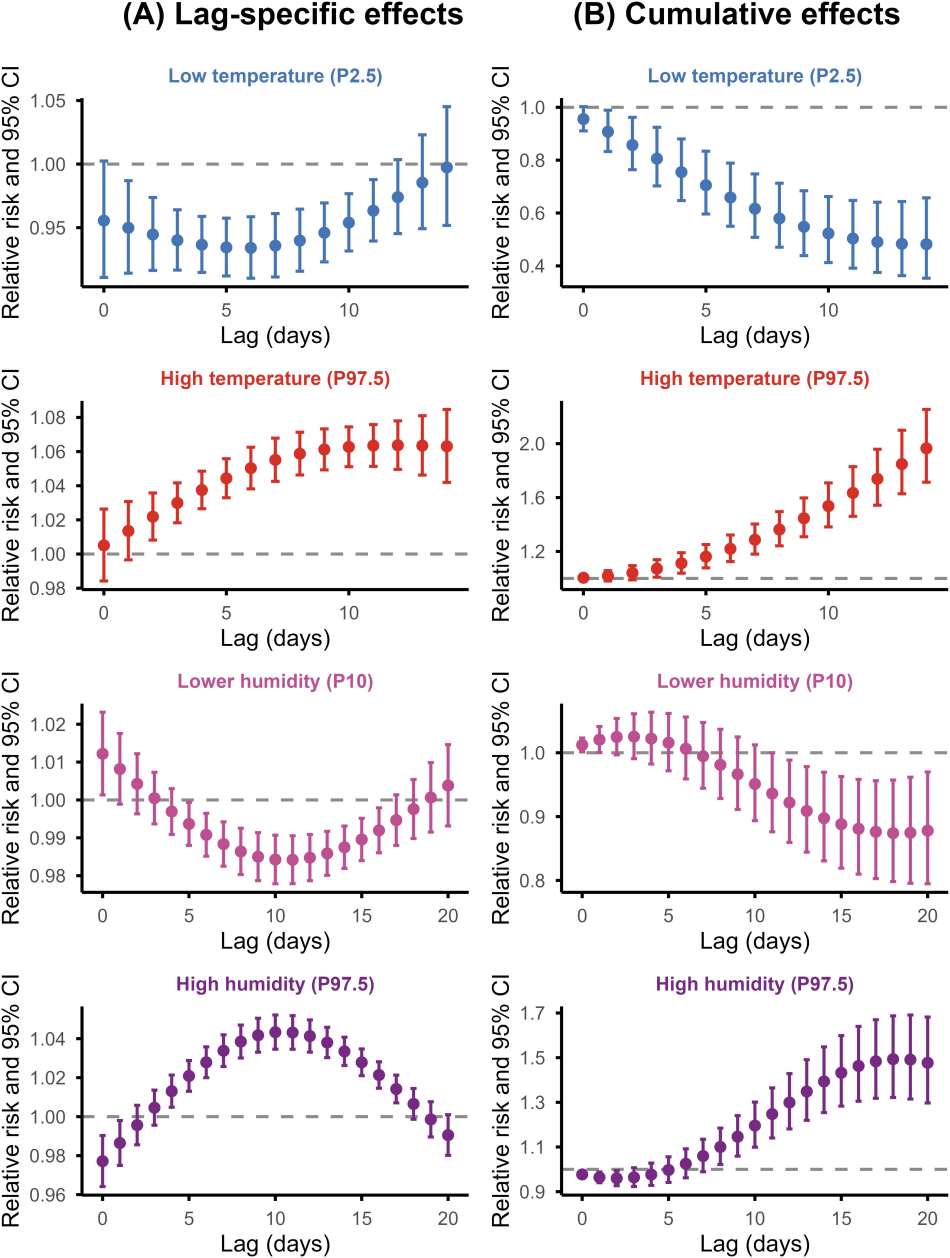
**(A)** lag-specific associations and **(B)** cumulative associations of temperature and relative humidity with daily outpatient visits for HA. Low and high temperature correspond to the 2.5th and 97.5th percentiles, respectively. Lower and high relative humidity correspond to the 10th and 97.5th percentiles, respectively. Points or lines indicate relative-risk estimates and error bars or shaded areas indicate 95% confidence intervals.

Figure 3A presents the lag-specific associations, and Figure 3B presents the cumulative associations for low temperature (P2.5), high temperature (P97.5), lower relative humidity (P10), and high relative humidity (P97.5). Points indicate RR estimates, vertical lines indicate 95% CIs, and the horizontal reference line indicates RR = 1. At the 97.5th percentile of temperature (30.63 °C), the lag-specific association was statistically significant from lag 2 through lag 14, with the 95% CIs above 1, and the highest point estimate occurred at lag 12. The cumulative RR over lags 0–14 was 1.966 (95% CI: 1.714–2.254). At the 2.5th percentile of temperature (10.77 °C), the 95% CIs were below 1 from lag 1 through lag 11, and the lowest point estimate occurred at lag 6. The cumulative RR over lags 0–14 was 0.482 (95% CI: 0.353–0.657). At the 97.5th percentile of relative humidity (93.21%), the 95% CIs were below 1 at lags 0–1 and above 1 from lag 4 through lag 17, with the highest point estimate at lag 10. The cumulative RR over lags 0–20 was 1.477 (95% CI: 1.297–1.682). At the 10th percentile of relative humidity (62.21%), the 95% CI was above 1 at lag 0 and below 1 from lag 5 through lag 16. The cumulative RR over lags 0–20 was 0.878 (95% CI: 0.795–0.970). The numerical estimates corresponding to the lag-specific and cumulative associations in Figure 3 are provided in Supplementary Table S3, Panels A and B.

### 3.5 Stratified associations by sex and age group

Table 3 presents cumulative associations by sex and age group. High temperature was associated with increased daily outpatient visits for HA among females (RR = 2.109, 95% CI: 1.761–2.526), males (RR = 1.871, 95% CI: 1.588–2.205), Children aged less than 36 months (RR = 1.982, 95% CI: 1.673–2.347), and children aged 36 months or older (RR = 1.965, 95% CI: 1.639–2.355). High relative humidity was also positively associated with outpatient visits in all four strata, with RRs ranging from 1.223 to 1.737.

**Table 3:** Cumulative associations of temperature and relative humidity with daily outpatient visits for HA, stratified by sex and age group.

Group	High temperature (P97.5)		Low temperature (P2.5)		High humidity (P97.5)		Lower humidity (P10)	
	RR	CI	RR	CI	RR	CI	RR	CI
Total outpatient visits	1.966	(1.714, 2.254)*	0.482	(0.353, 0.657)*	1.477	(1.297, 1.682)*	0.878	(0.795, 0.970)*
Female	2.109	(1.761, 2.526)*	0.371	(0.246, 0.559)*	1.555	(1.310, 1.846)*	0.846	(0.743, 0.964)*
Male	1.871	(1.588, 2.205)*	0.594	(0.410, 0.861)*	1.428	(1.222, 1.668)*	0.901	(0.799, 1.015)
<36 months	1.982	(1.673, 2.347)*	0.501	(0.352, 0.714)*	1.737	(1.489, 2.026)*	0.843	(0.747, 0.951)*
≥36 months	1.965	(1.639, 2.355)*	0.440	(0.279, 0.695)*	1.223	(1.022, 1.462)*	0.916	(0.801, 1.047)

RRs are cumulative estimates over lags 0–14 days for temperature and lags 0–20 days for relative humidity. Temperature estimates were centered at the median temperature (24.38 °C), and relative-humidity estimates were centered at the median relative humidity (79.15%). Age groups were defined as <36 months and ≥36 months. Stratum-specific estimates were descriptive; no formal interaction tests were performed.

* *P* < 0.05 indicates that the 95% CI excludes 1.

**Figure 4:**
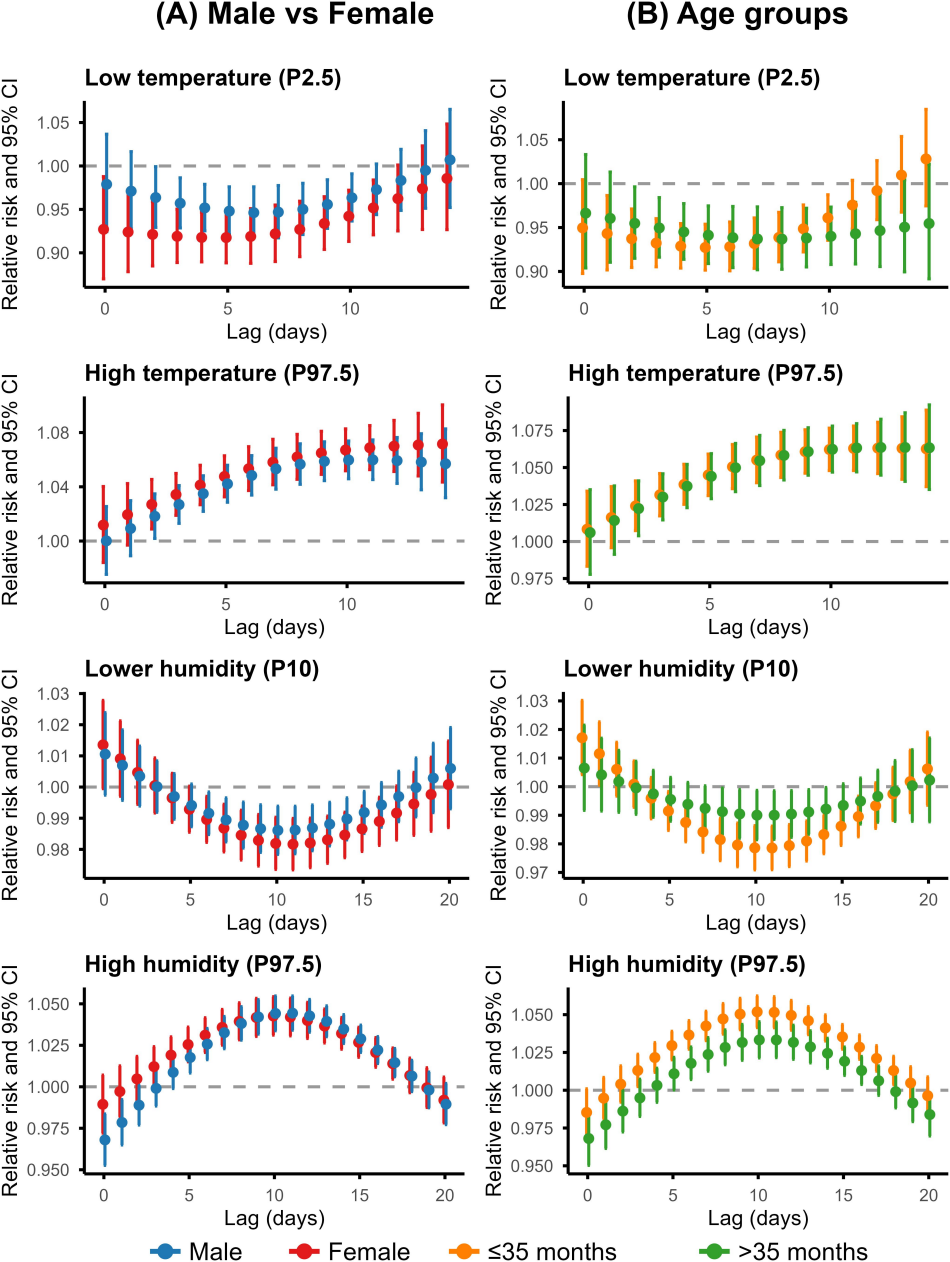
**(A)** sex-stratified and **(B)** age-stratified lag-response patterns of meteorological factors associated with daily outpatient visits for HA. Age groups were defined as younger than 36 and 36 months or older.

Lower relative humidity was inversely associated with outpatient visits among females (RR = 0.846, 95% CI: 0.743–0.964) and Children aged less than 36 months (RR = 0.843, 95% CI: 0.747–0.951), whereas the corresponding 95% CIs included 1 among males and children aged 36 months or older. These stratified results provided descriptive evidence on the numerical differences across demographic subgroups. Although the inverse association for lower relative humidity showed a larger point estimate among younger children than among older children, the estimates for the two age groups had overlapping confidence intervals. Figure 4 further illustrates the lag-response patterns across sex and age strata. The corresponding stratified lag-specific estimates are provided in Supplementary Table S3, Panels C and D. The curves were broadly similar between males and females and between the two age groups. High-temperature estimates generally increased across the lag period, whereas high-humidity estimates rose during intermediate lags and declined toward the end of the window. Lower-humidity estimates showed a transient inverse association during intermediate lags.

### 3.6 Sensitivity analysis

Across the 15 alternative temporal and seasonal control specifications, the low-temperature RR ranged from 0.304 to 0.596, the high-temperature RR ranged from 1.605 to 2.228, and the high-humidity RR ranged from 1.372 to 1.845; their 95% CIs excluded 1 in all 15 models. In contrast, the lower-humidity RR ranged from 0.824 to 1.040, with the 95% CI excluding 1 in only 5 of 15 models. These results indicate that the associations for low temperature, high temperature, and high relative humidity were robust to alternative temporal-control specifications, whereas the lower-humidity association was less stable.

In the separate-exposure models, the low-temperature estimate was similar to the mutually adjusted estimate (RR = 0.478, 95% CI: 0.359–0.636), whereas the high-temperature estimate was attenuated but remained statistically significant (RR = 1.450, 95% CI: 1.298–1.619). The lower-humidity estimate remained inverse (RR = 0.899, 95% CI: 0.817–0.990), while the high-humidity estimate was attenuated and was no longer statistically significant when temperature was omitted (RR = 1.054, 95% CI: 0.946–1.174). These changes suggest that mutual adjustment for temperature and relative humidity is important when estimating exposure-specific associations.

After excluding observations from the COVID-19 control period, associations remained statistically significant for low temperature (RR = 0.343, 95% CI: 0.244–0.481), high temperature (RR = 1.939, 95% CI: 1.673–2.246), and high relative humidity (RR = 1.605, 95% CI: 1.393–1.849). The lower-humidity estimate remained below 1 but was no longer statistically significant (RR = 0.965, 95% CI: 0.852–1.092). Full sensitivity results are provided in Supplementary Table S2.

## 4 Discussion

### 4.1 Meteorological factors (temperature and humidity) exhibit nonlinear and lagged associations with pediatric HA outpatient visits

Using a distributed lag nonlinear model, this study identified nonlinear and delayed associations between temperature, relative humidity, and daily outpatient visits for HA in Guangzhou. These findings provide quantitative evidence that meteorological conditions may be associated with temporal variation in HA outpatient visits in humid and warm regions. Although direct evidence on the meteorological associations of HA remains limited, our findings are generally consistent with previous observations that high temperature and humidity are associated with increased pediatric acute respiratory infections and enterovirus-related diseases (16, 17).

Previous studies on hand, foot, and mouth disease (HFMD), another common enterovirus-related childhood disease, have reported nonlinear and delayed associations with temperature. For example, temperature has been shown to have different directions of association across lag periods, with short-term inverse associations followed by positive associations during later lags (18). Coxsackievirus A is the predominant etiological group for HA, whereas EV71 is also a documented pathogen shared with HFMD (1, 19). Because both diseases can involve contact transmission, comparisons with HFMD may provide useful context (20). However, given differences in clinical manifestations, pathogens, and surveillance definitions between HA and HFMD, such comparisons should be interpreted cautiously.

From a biological and behavioral perspective, warm and humid environments may influence HA outpatient visits through several pathways. First, temperature and humidity may affect the survival and stability of enteroviruses, particularly Coxsackievirus A (e.g., serotypes A2, A4, A6, A10, A16), which are more commonly associated with HA than EV71, on surfaces and in aerosols (21). In our study, the detection rates were as follows: A6 in 209/363 (57.6%), A16 in 123/449 (27.4%), A10 in 80/359 (22.3%), and EV71 in only 43/451 (9.5%). Second, humid and warm weather may influence children's activity patterns and indoor crowding, thereby increasing opportunities for transmission through respiratory droplets and close contact (22). These mechanisms are also consistent with evidence that group settings, such as kindergarten attendance and household child contacts, are associated with enterovirus infection risk (23).

After entering the human body, enteroviruses can replicate in mucosal tissues of the upper respiratory and alimentary tracts (24), triggering local inflammatory responses and epithelial injury. Clinically, this process can manifest as fever, sore throat, oral vesicles, and ulcers, sometimes accompanied by upper respiratory symptoms such as cough and rhinorrhea (25–27). The delayed associations observed in this study may therefore reflect a combination of environmental effects on viral persistence, contact patterns, incubation, symptom development, and subsequent healthcare-seeking behavior. In addition, children's immature immune systems and relatively weaker hygiene awareness may increase their susceptibility to infections in group settings such as daycare centers (22). The seasonal pattern observed in this study is also consistent with reports from humid and warm regions in Asia, including Japan and Malaysia, where HA or related enterovirus infections show higher activity during warmer seasons (28, 29). Together, these findings support the relevance of meteorological factors in HA surveillance, while emphasizing that the observed associations should be interpreted as population-level temporal associations rather than direct causal effects.

### 4.2 High temperature and high humidity are associated with increased HA outpatient visits, with persistent and delayed patterns

High temperature and high humidity were key meteorological conditions associated with increased daily outpatient visits for HA, and they showed distinct delayed patterns. This finding has practical implications for medical institutions in terms of surveillance and resource preparedness during high-incidence seasons. During humid and hot periods, healthcare providers should anticipate lagged increases in pediatric outpatient demand. In the present study, high-temperature associations were observed mainly over lags 2–14 days, with the highest lag-specific point estimate at lag 12. In contrast, high-humidity associations were observed mainly from lag 4 to lag 17, suggesting a delayed and relatively prolonged pattern. These results indicate that preparedness should not be limited to the day of exposure or the immediate few days afterward, but should consider extended response windows.

By implementing time-sensitive management informed by delayed meteorological associations, healthcare institutions may move from general “peak season alerts” toward more actionable phased preparedness. Such measures may include closer surveillance of HA outpatient visits, flexible adjustment of pediatric outpatient resources, and preparation of relevant clinical supplies during periods following humid and hot weather. In addition, studies conducted in different regions have shown that high temperature is associated with increased incidence of gastrointestinal and enterovirus-related diseases, including HFMD, which supports the relevance of meteorological conditions to pediatric enterovirus activity (30). Therefore, incorporating the lagged and persistent associations of meteorological factors into surveillance may provide a useful basis for regional child health preparedness strategies (31).

### 4.3 Low temperature is robustly associated with fewer HA outpatient visits

Low temperature was associated with fewer HA outpatient visits, with a cumulative RR of 0.482 over lags 0–14 days. This inverse association was robust across sensitivity analyses, with the 95% CI excluding 1 in all 15 alternative temporal-control models and remaining significant after excluding the COVID-19 period. This inverse association appeared early (from lag 1) and persisted through lag 11. Reduced viral survival at lower temperatures, decreased outdoor activity, and altered host susceptibility may contribute to this pattern. These findings indicate that cold conditions may be associated with reduced HA transmission or fewer HA-related outpatient visits, although the underlying mechanisms require further investigation.

### 4.4 Age-stratified patterns in the associations with relative humidity

In the age-stratified analysis, the inverse association between lower relative humidity and daily outpatient visits for HA was statistically significant in children aged less than 36 months, whereas the 95% CI included 1 among older children. However, the estimates for the two age groups were in the same direction and had overlapping confidence intervals. In addition, the lower-humidity association was less stable in sensitivity analyses. No formal interaction tests were performed; therefore, this age-stratified pattern should be interpreted as descriptive evidence of numerical differences rather than evidence of effect modification or differential susceptibility by age.

Several developmental and behavioral factors could in principle contribute to numerical differences in stratified estimates, although formal interaction tests were not performed. These include developing immune systems and less mature immune memory (32, 33), narrower airways and less mature mucociliary clearance (34–36), as well as frequent hand-mouth behaviors (37–39). Such characteristics may increase susceptibility to enterovirus infections, particularly in group settings like daycare centers where close contact facilitates transmission (23).

Previous studies have also suggested that younger age is associated with higher susceptibility to enterovirus-related diseases such as HFMD or HA (23). However, in the present study, the stratified results should not be interpreted as confirming age-related effect modification. Rather, they suggest that age may be a relevant factor to consider in future multicenter studies with larger sample sizes, more refined age categories, and formal evaluation of subgroup differences.

## 5 Strengths of this study

This study systematically quantified the associations of temperature and relative humidity with daily outpatient visits for HA in Guangzhou, based on a 10-year dataset of 83,146 outpatient visits collected from 2014 to 2023. The long-term time-series data allowed us to characterize the seasonal clustering of HA outpatient visits in Guangzhou and to evaluate delayed associations across multiple epidemic seasons. Methodologically, this study applied a distributed lag nonlinear model (DLNM), which enabled simultaneous assessment of nonlinear exposure-response associations and lag-response patterns. Temperature, relative humidity, and PM_2.5_ were simultaneously included using cross-basis functions, allowing the temperature and humidity estimates to be mutually adjusted and further adjusted for PM_2.5_.

These findings have practical implications for pediatric infectious-disease surveillance and healthcare preparedness. High temperature and high relative humidity were consistently associated with increased daily outpatient visits for HA, and these associations showed delayed patterns, which is broadly consistent with previous domestic evidence (6). During periods of high HA activity, meteorological information may help inform timely surveillance and preparedness planning. In addition, the stratified analyses by sex and age group provided descriptive evidence that the observed meteorological associations were generally consistent across demographic subgroups, which may be useful for future multicenter studies and the development of refined early-warning models.

## 6 Limitations of the study

This study has several limitations. First, the visit data were obtained from a single provincial maternal and child hospital, which may not fully represent the city-wide disease spectrum of HA, particularly mild cases managed in community clinics or other healthcare institutions. Therefore, the findings should be interpreted as reflecting hospital-based outpatient visits rather than the overall incidence of HA in Guangzhou. Second, this ecological time-series study can evaluate temporal associations at the population level but cannot establish individual-level causal relationships. Third, meteorological and pollutant data were based on city-wide daily averages, which did not account for spatial heterogeneity in environmental exposures across different districts of Guangzhou. This may have introduced exposure-measurement error and biased the estimated associations. Fourth, although PM_2.5_ was included as an air-pollution covariate, other air pollutants were unavailable and could not be adjusted for, leaving the possibility of residual confounding. Fifth, the model could not adjust for individual-level or behavioral factors, such as school or daycare schedules, hygiene practices, population mobility, household contact patterns, and healthcare-seeking preferences; therefore, residual confounding from these unmeasured factors cannot be excluded. These factors may influence children's exposure frequency, transmission opportunities, and visit patterns, thereby affecting the estimation of meteorological associations. Sixth, stratified analyses were limited to broad sex and age groups, and subgroup patterns should therefore be interpreted descriptively rather than as definitive evidence of effect modification. Further stratification into narrower age intervals (e.g., 0–12, 13–24, 25–36 months) or season-specific analyses was not performed due to sample size and power considerations, which may have obscured more detailed age- or season-dependent patterns. Finally, although the COVID-19 control period was adjusted for and further examined in a sensitivity analysis, residual influence from pandemic-related changes in healthcare-seeking behavior, social contact patterns, and the circulation of pediatric infectious diseases cannot be completely excluded.

Future studies should integrate multicenter surveillance data, high-resolution environmental exposure assessment, and mobility or behavioral data to improve exposure measurement and confounder control. Such efforts would help refine HA risk prediction models and support more precise meteorology-informed surveillance and preparedness strategies.

## 7 Conclusion

This time-series study found nonlinear and delayed associations between temperature, relative humidity, and daily outpatient visits for HA in Guangzhou. High temperature and high relative humidity were associated with increased outpatient visits, with cumulative associations observed over lags 0–14 days for temperature and 0–20 days for relative humidity. Low temperature and lower relative humidity were associated with fewer outpatient visits in the main model; however, the lower-humidity association was less stable in sensitivity analyses and should therefore be interpreted cautiously. Stratified analyses showed broadly similar patterns across sex and age groups, providing descriptive evidence on the numerical estimates across demographic subgroups. No formal interaction tests were performed. These findings support the use of meteorological information in HA surveillance and preparedness in humid-heat regions. Future studies should integrate multicenter data, high-resolution environmental exposure assessment, and behavioral variables to improve exposure measurement, confounder control, and risk prediction.

## Data Availability

The raw data supporting the conclusions of this article will be made available by the authors, without undue reservation.
