# Supplementary material for: Lag effects of meteorological factors on the risk of daily outpatient visits for herpangina in Guangzhou (2014–2023)
Source: Front Pediatr. 2026 Aug 4;14:1868116. doi: 10.3389/fped.2026.1868116 (PMC13481785; doi:10.3389/fped.2026.1868116)
Supplement: Supplementary file 1 [file Datasheet1.pdf]

Supplementary Material

1 Supplementary Data

2 Supplementary Figures and Tables

2.1 Supplementary Tables

Supplementary Table S1. Comparison of candidate lag windows and spline degrees of freedom for temperature and relative humidity.

Candidate parameter	Value	Q-AIC	Delta Q-AIC	Low temperature RR (95% CI)	High temperature RR (95% CI)	Lower humidity RR (95% CI)	High humidity RR (95% CI)	Temperature boundary RR (95% CI)
Temperature maximum lag	7	13722.9	118.0	0.575 (0.468-0.707)	1.184 (1.082-1.296)	0.926 (0.840-1.022)	1.151 (1.021-1.296)	1.073 (1.043-1.105)
Temperature maximum lag	10	13692.7	87.9	0.555 (0.432-0.713)	1.427 (1.279-1.593)	0.904 (0.819-0.997)	1.283 (1.133-1.453)	1.065 (1.040-1.091)
**Temperature maximum lag**	**14**	**13636.9**	**32.0**	**0.458 (0.334-0.629)**	**1.972 (1.720-2.262)**	**0.881 (0.797-0.973)**	**1.489 (1.307-1.696)**	**1.064 (1.042-1.085)**
Temperature maximum lag	21	13607.1	2.2	0.424 (0.281-0.640)	2.981 (2.464-3.607)	0.856 (0.772-0.950)	1.733 (1.508-1.990)	1.018 (1.001-1.036)
Temperature maximum lag	24	13605.9	1.0	0.358 (0.228-0.561)	3.037 (2.480-3.720)	0.867 (0.781-0.962)	1.744 (1.519-2.002)	0.993 (0.979-1.008)
Temperature maximum lag	25	13604.9	0.0	0.346 (0.218-0.549)	3.039 (2.470-3.739)	0.867 (0.781-0.962)	1.743 (1.518-2.001)	0.988 (0.974-1.002)
Humidity maximum lag	10	13646.2	25.9	0.437 (0.320-0.597)	1.632 (1.441-1.848)	0.967 (0.906-1.032)	1.138 (1.049-1.235)	1.015 (0.996-1.035)
Humidity maximum lag	14	13620.4	0.0	0.432 (0.314-0.594)	1.892 (1.651-2.169)	0.930 (0.857-1.008)	1.353 (1.217-1.503)	1.047 (1.023-1.071)
**Humidity maximum lag**	**20**	**13636.9**	**16.5**	**0.458 (0.334-0.629)**	**1.972 (1.720-2.262)**	**0.881 (0.797-0.973)**	**1.489 (1.307-1.696)**	**1.064 (1.042-1.085)**
Humidity maximum lag	21	13644.3	23.9	0.457 (0.333-0.627)	1.966 (1.714-2.255)	0.873 (0.788-0.968)	1.501 (1.312-1.717)	1.065 (1.044-1.087)
Humidity maximum lag	28	13696.9	76.6	0.444 (0.323-0.609)	1.761 (1.537-2.019)	0.846 (0.744-0.960)	1.711 (1.445-2.025)	1.050 (1.029-1.071)
Temperature exposure df	2	13638.6	1.8	0.482 (0.352-0.659)	1.875 (1.655-2.126)	0.889 (0.805-0.982)	1.422 (1.261-1.605)	1.057 (1.037-1.078)
**Temperature exposure df**	**3**	**13636.9**	**0.1**	**0.458 (0.334-0.629)**	**1.972 (1.720-2.262)**	**0.881 (0.797-0.973)**	**1.489 (1.307-1.696)**	**1.064 (1.042-1.085)**
Temperature exposure df	4	13636.8	0.0	0.439 (0.318-0.605)	2.008 (1.743-2.313)	0.885 (0.801-0.978)	1.491 (1.310-1.698)	1.061 (1.039-1.083)
Temperature lag df	2	13643.0	6.1	0.437 (0.321-0.595)	2.023 (1.766-2.317)	0.886 (0.802-0.979)	1.489 (1.307-1.696)	1.077 (1.060-1.093)
**Temperature lag df**	**3**	**13636.9**	**0.0**	**0.458 (0.334-0.629)**	**1.972 (1.720-2.262)**	**0.881 (0.797-0.973)**	**1.489 (1.307-1.696)**	**1.064 (1.042-1.085)**
Temperature lag df	4	13638.6	1.7	0.453 (0.330-0.622)	1.958 (1.707-2.247)	0.881 (0.797-0.973)	1.495 (1.312-1.703)	1.070 (1.043-1.097)
**Humidity exposure df**	**2**	**13636.9**	**23.8**	**0.458 (0.334-0.629)**	**1.972 (1.720-2.262)**	**0.881 (0.797-0.973)**	**1.489 (1.307-1.696)**	**1.064 (1.042-1.085)**
Humidity exposure df	3	13615.1	2.0	0.450 (0.328-0.617)	2.040 (1.777-2.343)	0.956 (0.837-1.092)	1.609 (1.379-1.877)	1.070 (1.048-1.091)
Humidity exposure df	4	13613.1	0.0	0.452 (0.330-0.620)	1.949 (1.679-2.263)	0.903 (0.732-1.114)	1.551 (1.321-1.822)	1.067 (1.045-1.089)
Humidity lag df	2	13721.5	105.3	0.449 (0.327-0.617)	1.757 (1.534-2.013)	0.889 (0.804-0.983)	1.448 (1.270-1.652)	1.044 (1.023-1.064)
**Humidity lag df**	**3**	**13636.9**	**20.7**	**0.458 (0.334-0.629)**	**1.972 (1.720-2.262)**	**0.881 (0.797-0.973)**	**1.489 (1.307-1.696)**	**1.064 (1.042-1.085)**
Humidity lag df	4	13616.2	0.0	0.426 (0.310-0.586)	1.936 (1.688-2.221)	0.888 (0.804-0.981)	1.479 (1.298-1.684)	1.047 (1.025-1.071)

Candidate models were fitted using a common analysis period beginning on January 29, 2014, because the longest candidate lag window was 28 days and exposure data before January 1, 2014 were unavailable. Using the same analysis period ensured that Q-AIC values were comparable across candidate models. The primary model, whose longest lag window was 20 days, used all available observations beginning on January 21, 2014. Consequently, the RR estimates in this table may differ slightly from the primary estimates. Model selection considered Q-AIC and the stability of the estimated associations. Temperature boundary RR denotes the lag-specific RR at the final day of the candidate temperature lag window, evaluated at P97.5 relative to the median temperature.

Supplementary Table S2. Sensitivity analyses of the cumulative associations of temperature and relative humidity with daily outpatient visits for herpangina.

Panel A. Alternative specifications for long-term and seasonal control.

Time df/year	Seasonal k	Total time df	Low temperature RR (95% CI)	High temperature RR (95% CI)	Lower humidity RR (95% CI)	High humidity RR (95% CI)
6	6	60	0.464 (0.344-0.627)	2.195 (1.932-2.495)	0.948 (0.863-1.041)	1.504 (1.346-1.681)
7	6	70	0.373 (0.272-0.510)	2.147 (1.870-2.464)	1.025 (0.928-1.133)	1.845 (1.635-2.082)
8	6	80	0.409 (0.301-0.555)	1.946 (1.699-2.229)	1.005 (0.912-1.107)	1.420 (1.261-1.599)
9	6	90	0.355 (0.261-0.483)	2.007 (1.759-2.289)	0.891 (0.807-0.983)	1.611 (1.433-1.811)
10	6	100	0.596 (0.434-0.819)	1.760 (1.531-2.023)	0.824 (0.744-0.913)	1.372 (1.202-1.567)
6	8	60	0.393 (0.292-0.530)	2.228 (1.959-2.534)	0.972 (0.887-1.065)	1.534 (1.371-1.716)
7	8	70	0.472 (0.342-0.651)	1.605 (1.395-1.848)	1.040 (0.938-1.152)	1.572 (1.385-1.785)
8	8	80	0.422 (0.311-0.572)	1.946 (1.700-2.227)	1.002 (0.909-1.103)	1.465 (1.300-1.651)

Time df/year	Seasonal k	Total time df	Low temperature RR (95% CI)	High temperature RR (95% CI)	Lower humidity RR (95% CI)	High humidity RR (95% CI)
9	8	90	0.304 (0.223-0.415)	1.959 (1.719-2.233)	0.921 (0.836-1.016)	1.521 (1.353-1.709)
10	8	100	0.482 (0.353-0.657)	1.966 (1.714-2.254)	0.878 (0.795-0.970)	1.477 (1.297-1.682)
6	10	60	0.437 (0.323-0.590)	2.218 (1.948-2.524)	0.973 (0.885-1.069)	1.538 (1.374-1.722)
7	10	70	0.341 (0.248-0.468)	2.043 (1.773-2.353)	1.001 (0.904-1.109)	1.768 (1.564-1.997)
8	10	80	0.440 (0.324-0.598)	1.970 (1.722-2.255)	0.950 (0.861-1.047)	1.460 (1.296-1.645)
9	10	90	0.372 (0.270-0.510)	1.886 (1.639-2.170)	0.828 (0.747-0.917)	1.522 (1.348-1.719)
10	10	100	0.517 (0.379-0.707)	1.867 (1.626-2.143)	0.885 (0.800-0.979)	1.454 (1.276-1.657)

Long-term temporal control was varied from 6 to 10 df per year, and the cyclic seasonal spline was evaluated at k = 6, 8, and 10. Values are cumulative RRs (95% CIs).

Panel B. Primary analysis and analysis excluding the COVID-19 control period.

Analysis	Low temperature (P2.5)	High temperature (P97.5)	Lower humidity (P10)	High humidity (P97.5)
Main model	0.482 (0.353-0.657)	1.966 (1.714-2.254)	0.878 (0.795-0.970)	1.477 (1.297-1.682)
Excluding COVID-19 period	0.343 (0.244-0.481)	1.939 (1.673-2.246)	0.965 (0.852-1.092)	1.605 (1.393-1.849)

The COVID-19 control period was defined as January 23, 2020 to December 31, 2022. In the exclusion analysis, the pre-COVID and post-COVID observations were treated as independent time-series segments.

Panel C. Mutually adjusted and separate-exposure models.

Exposure contrast	Mutually adjusted model, RR (95% CI)	Separate-exposure model, RR (95% CI)	Change without mutual adjustment (%)
Low temperature (P2.5)	0.482 (0.353-0.657)	0.478 (0.359-0.636)	-0.9
High temperature (P97.5)	1.966 (1.714-2.254)	1.450 (1.298-1.619)	-26.2
Lower humidity (P10)	0.878 (0.795-0.970)	0.899 (0.817-0.990)	2.4
High humidity (P97.5)	1.477 (1.297-1.682)	1.054 (0.946-1.174)	-28.6

The mutually adjusted model simultaneously included temperature, relative humidity, and $PM_{2.5}$ cross-basis terms. Each separate-exposure model omitted the other meteorological exposure but retained $PM_{2.5}$ and all temporal covariates. Low and high temperature correspond to P2.5 and P97.5; lower and high relative humidity correspond to P10 and P97.5. Abbreviations: CI, confidence interval; RR, relative risk.

Supplementary Table S3. Lag-specific and cumulative relative risks for the associations shown in Figures 3 and 4.

Panel A. Overall lag-specific relative risks corresponding to Figure 3A.

Lag (days)	Low temperature (P2.5)	High temperature (P97.5)	Lower humidity (P10)	High humidity (P97.5)
0	0.956 (0.911-1.002)	1.005 (0.984-1.026)	1.012 (1.001-1.023)	0.977 (0.964-0.990)
1	0.950 (0.914-0.987)	1.014 (0.997-1.031)	1.008 (0.999-1.018)	0.986 (0.975-0.998)
2	0.945 (0.916-0.974)	1.022 (1.008-1.036)	1.004 (0.996-1.012)	0.996 (0.986-1.006)
3	0.940 (0.917-0.964)	1.030 (1.018-1.042)	1.000 (0.994-1.007)	1.005 (0.996-1.014)
4	0.937 (0.915-0.959)	1.037 (1.027-1.049)	0.997 (0.991-1.003)	1.013 (1.005-1.021)
5	0.934 (0.912-0.957)	1.044 (1.033-1.056)	0.994 (0.988-0.999)	1.021 (1.013-1.029)
6	0.934 (0.910-0.959)	1.050 (1.038-1.063)	0.991 (0.985-0.996)	1.028 (1.020-1.036)
7	0.936 (0.911-0.961)	1.055 (1.043-1.068)	0.988 (0.983-0.994)	1.034 (1.026-1.042)
8	0.940 (0.916-0.965)	1.059 (1.046-1.071)	0.986 (0.980-0.993)	1.038 (1.030-1.047)
9	0.946 (0.923-0.969)	1.061 (1.049-1.073)	0.985 (0.979-0.991)	1.042 (1.033-1.050)
10	0.954 (0.932-0.977)	1.063 (1.051-1.074)	0.984 (0.978-0.991)	1.043 (1.035-1.052)
11	0.963 (0.939-0.988)	1.063 (1.051-1.076)	0.984 (0.978-0.991)	1.043 (1.035-1.052)
12	0.974 (0.945-1.003)	1.064 (1.050-1.078)	0.985 (0.979-0.991)	1.041 (1.033-1.050)
13	0.985 (0.949-1.023)	1.063 (1.046-1.081)	0.986 (0.980-0.992)	1.038 (1.030-1.046)
14	0.997 (0.952-1.045)	1.063 (1.042-1.085)	0.988 (0.982-0.993)	1.033 (1.026-1.041)
15	—	—	0.990 (0.984-0.995)	1.028 (1.021-1.035)
16	—	—	0.992 (0.986-0.998)	1.021 (1.015-1.028)
17	—	—	0.995 (0.988-1.001)	1.014 (1.007-1.021)

Lag (days)	Low temperature (P2.5)	High temperature (P97.5)	Lower humidity (P10)	High humidity (P97.5)
18	—	—	0.998 (0.990-1.005)	1.007 (0.999-1.014)
19	—	—	1.001 (0.992-1.010)	0.999 (0.990-1.008)
20	—	—	1.004 (0.993-1.015)	0.991 (0.980-1.001)

Values are RR (95% CI). Temperature was evaluated over lags 0-14 days and relative humidity over lags 0-20 days; em dashes indicate lags outside the prespecified temperature window.

Panel B. Overall cumulative relative risks corresponding to Figure 3B.

Lag (days)	Low temperature (P2.5)	High temperature (P97.5)	Lower humidity (P10)	High humidity (P97.5)
0	0.956 (0.911-1.002)	1.005 (0.984-1.026)	1.012 (1.001-1.023)	0.977 (0.964-0.990)
1	0.908 (0.833-0.989)	1.019 (0.981-1.058)	1.020 (1.000-1.041)	0.964 (0.940-0.988)
2	0.857 (0.764-0.962)	1.041 (0.989-1.095)	1.025 (0.997-1.054)	0.960 (0.927-0.994)
3	0.806 (0.703-0.924)	1.072 (1.009-1.139)	1.025 (0.991-1.061)	0.964 (0.923-1.007)
4	0.755 (0.647-0.880)	1.112 (1.039-1.191)	1.022 (0.982-1.063)	0.977 (0.928-1.028)
5	0.705 (0.596-0.834)	1.162 (1.078-1.252)	1.016 (0.972-1.062)	0.997 (0.941-1.056)
6	0.659 (0.550-0.789)	1.220 (1.125-1.322)	1.006 (0.959-1.056)	1.025 (0.962-1.092)
7	0.616 (0.508-0.748)	1.287 (1.180-1.404)	0.995 (0.944-1.047)	1.059 (0.989-1.135)
8	0.579 (0.471-0.713)	1.363 (1.242-1.496)	0.981 (0.928-1.037)	1.100 (1.022-1.185)
9	0.548 (0.439-0.684)	1.446 (1.309-1.598)	0.966 (0.911-1.025)	1.146 (1.059-1.241)
10	0.523 (0.412-0.662)	1.537 (1.382-1.709)	0.951 (0.894-1.012)	1.196 (1.099-1.301)
11	0.504 (0.391-0.648)	1.634 (1.461-1.829)	0.936 (0.876-1.000)	1.247 (1.140-1.365)
12	0.490 (0.375-0.641)	1.739 (1.543-1.959)	0.922 (0.860-0.989)	1.299 (1.181-1.429)
13	0.483 (0.363-0.643)	1.849 (1.628-2.099)	0.909 (0.844-0.978)	1.348 (1.220-1.491)
14	0.482 (0.353-0.657)	1.966 (1.714-2.254)	0.897 (0.831-0.970)	1.393 (1.254-1.548)
15	—	—	0.888 (0.819-0.963)	1.432 (1.283-1.599)
16	—	—	0.881 (0.810-0.958)	1.463 (1.305-1.640)
17	—	—	0.876 (0.803-0.956)	1.484 (1.318-1.670)
18	—	—	0.874 (0.798-0.957)	1.493 (1.322-1.687)
19	—	—	0.875 (0.796-0.962)	1.491 (1.315-1.691)
20	—	—	0.878 (0.795-0.970)	1.477 (1.297-1.682)

Each estimate is cumulative from lag 0 through the indicated lag. Values are RR (95% CI).

Panel C. Sex-stratified lag-specific relative risks corresponding to Figure 4A.

Exposure contrast	Lag (days)	Female	Male
Low temperature (P2.5)	0	0.927 (0.870-0.988)	0.979 (0.924-1.037)
Low temperature (P2.5)	1	0.924 (0.878-0.972)	0.971 (0.928-1.016)
Low temperature (P2.5)	2	0.921 (0.885-0.959)	0.964 (0.929-0.999)
Low temperature (P2.5)	3	0.919 (0.889-0.950)	0.957 (0.929-0.986)
Low temperature (P2.5)	4	0.918 (0.890-0.947)	0.952 (0.925-0.979)
Low temperature (P2.5)	5	0.918 (0.888-0.948)	0.948 (0.921-0.976)
Low temperature (P2.5)	6	0.919 (0.888-0.951)	0.946 (0.917-0.976)
Low temperature (P2.5)	7	0.922 (0.890-0.955)	0.947 (0.917-0.977)
Low temperature (P2.5)	8	0.927 (0.896-0.959)	0.950 (0.921-0.980)
Low temperature (P2.5)	9	0.934 (0.904-0.965)	0.956 (0.928-0.984)
Low temperature (P2.5)	10	0.942 (0.913-0.972)	0.963 (0.937-0.991)
Low temperature (P2.5)	11	0.952 (0.921-0.984)	0.973 (0.944-1.002)
Low temperature (P2.5)	12	0.962 (0.925-1.001)	0.983 (0.949-1.019)
Low temperature (P2.5)	13	0.974 (0.927-1.023)	0.995 (0.951-1.041)
Low temperature (P2.5)	14	0.986 (0.927-1.048)	1.007 (0.952-1.065)
High temperature (P97.5)	0	1.012 (0.984-1.040)	1.000 (0.975-1.025)
High temperature (P97.5)	1	1.019 (0.997-1.042)	1.009 (0.989-1.030)
High temperature (P97.5)	2	1.027 (1.009-1.045)	1.018 (1.002-1.035)
High temperature (P97.5)	3	1.034 (1.019-1.050)	1.027 (1.013-1.041)
High temperature (P97.5)	4	1.041 (1.027-1.056)	1.035 (1.022-1.048)
High temperature (P97.5)	5	1.048 (1.033-1.063)	1.042 (1.029-1.056)
High temperature (P97.5)	6	1.053 (1.037-1.070)	1.048 (1.034-1.063)
High temperature (P97.5)	7	1.058 (1.041-1.075)	1.053 (1.038-1.068)
High temperature (P97.5)	8	1.062 (1.046-1.079)	1.057 (1.042-1.072)
High temperature (P97.5)	9	1.065 (1.049-1.081)	1.059 (1.045-1.073)
High temperature (P97.5)	10	1.067 (1.052-1.083)	1.060 (1.046-1.074)
High temperature (P97.5)	11	1.069 (1.053-1.085)	1.060 (1.045-1.075)
High temperature (P97.5)	12	1.070 (1.051-1.089)	1.059 (1.042-1.076)
High temperature (P97.5)	13	1.071 (1.048-1.094)	1.058 (1.038-1.079)
High temperature (P97.5)	14	1.072 (1.043-1.100)	1.057 (1.032-1.083)
Lower humidity (P10)	0	1.014 (0.999-1.028)	1.011 (0.997-1.024)
Lower humidity (P10)	1	1.009 (0.997-1.021)	1.007 (0.996-1.018)
Lower humidity (P10)	2	1.005 (0.994-1.015)	1.003 (0.994-1.013)
Lower humidity (P10)	3	1.000 (0.992-1.009)	1.000 (0.992-1.008)
Lower humidity (P10)	4	0.997 (0.989-1.004)	0.997 (0.990-1.004)
Lower humidity (P10)	5	0.993 (0.985-1.000)	0.994 (0.987-1.001)
Lower humidity (P10)	6	0.990 (0.982-0.997)	0.992 (0.985-0.998)

Exposure contrast	Lag (days)	Female	Male
Lower humidity (P10)	7	0.987 (0.979-0.994)	0.989 (0.982-0.997)
Lower humidity (P10)	8	0.985 (0.977-0.993)	0.988 (0.980-0.995)
Lower humidity (P10)	9	0.983 (0.975-0.991)	0.987 (0.979-0.994)
Lower humidity (P10)	10	0.982 (0.974-0.990)	0.986 (0.978-0.994)
Lower humidity (P10)	11	0.982 (0.973-0.990)	0.986 (0.979-0.994)
Lower humidity (P10)	12	0.982 (0.974-0.990)	0.987 (0.980-0.994)
Lower humidity (P10)	13	0.983 (0.975-0.991)	0.988 (0.981-0.995)
Lower humidity (P10)	14	0.985 (0.977-0.992)	0.990 (0.983-0.997)
Lower humidity (P10)	15	0.987 (0.979-0.994)	0.992 (0.985-0.999)
Lower humidity (P10)	16	0.989 (0.981-0.997)	0.994 (0.987-1.001)
Lower humidity (P10)	17	0.992 (0.983-1.000)	0.997 (0.989-1.005)
Lower humidity (P10)	18	0.995 (0.984-1.005)	1.000 (0.990-1.009)
Lower humidity (P10)	19	0.998 (0.986-1.010)	1.003 (0.992-1.014)
Lower humidity (P10)	20	1.001 (0.987-1.015)	1.006 (0.993-1.019)
High humidity (P97.5)	0	0.989 (0.972-1.007)	0.968 (0.953-0.984)
High humidity (P97.5)	1	0.997 (0.982-1.013)	0.978 (0.965-0.992)
High humidity (P97.5)	2	1.005 (0.991-1.018)	0.989 (0.977-1.001)
High humidity (P97.5)	3	1.012 (1.000-1.024)	0.999 (0.988-1.010)
High humidity (P97.5)	4	1.019 (1.008-1.030)	1.009 (0.999-1.019)
High humidity (P97.5)	5	1.025 (1.015-1.036)	1.018 (1.008-1.027)
High humidity (P97.5)	6	1.031 (1.021-1.042)	1.026 (1.016-1.035)
High humidity (P97.5)	7	1.036 (1.025-1.047)	1.033 (1.023-1.042)
High humidity (P97.5)	8	1.039 (1.028-1.051)	1.038 (1.028-1.048)
High humidity (P97.5)	9	1.042 (1.030-1.053)	1.042 (1.032-1.053)
High humidity (P97.5)	10	1.043 (1.031-1.054)	1.044 (1.034-1.055)
High humidity (P97.5)	11	1.042 (1.031-1.053)	1.044 (1.034-1.055)
High humidity (P97.5)	12	1.040 (1.029-1.051)	1.043 (1.033-1.053)
High humidity (P97.5)	13	1.037 (1.026-1.047)	1.039 (1.030-1.049)
High humidity (P97.5)	14	1.032 (1.023-1.042)	1.035 (1.026-1.043)
High humidity (P97.5)	15	1.027 (1.018-1.036)	1.029 (1.021-1.037)
High humidity (P97.5)	16	1.021 (1.012-1.030)	1.022 (1.014-1.030)
High humidity (P97.5)	17	1.014 (1.005-1.023)	1.015 (1.006-1.023)
High humidity (P97.5)	18	1.007 (0.996-1.017)	1.006 (0.997-1.016)
High humidity (P97.5)	19	0.999 (0.988-1.011)	0.998 (0.987-1.009)
High humidity (P97.5)	20	0.992 (0.978-1.006)	0.990 (0.977-1.002)

Values are RR (95% CI). No formal interaction test was performed.

Panel D. Age-stratified lag-specific relative risks corresponding to Figure 4B.

Exposure contrast	Lag (days)	≤35 months	>35 months
Low temperature (P2.5)	0	0.950 (0.898-1.005)	0.966 (0.904-1.033)
Low temperature (P2.5)	1	0.943 (0.902-0.986)	0.960 (0.910-1.013)
Low temperature (P2.5)	2	0.937 (0.905-0.971)	0.955 (0.915-0.996)
Low temperature (P2.5)	3	0.932 (0.905-0.960)	0.950 (0.916-0.984)
Low temperature (P2.5)	4	0.929 (0.904-0.955)	0.945 (0.914-0.977)
Low temperature (P2.5)	5	0.927 (0.902-0.954)	0.941 (0.909-0.975)
Low temperature (P2.5)	6	0.928 (0.901-0.956)	0.939 (0.904-0.974)
Low temperature (P2.5)	7	0.932 (0.904-0.961)	0.937 (0.902-0.974)
Low temperature (P2.5)	8	0.939 (0.911-0.967)	0.937 (0.902-0.973)
Low temperature (P2.5)	9	0.949 (0.922-0.976)	0.938 (0.905-0.972)
Low temperature (P2.5)	10	0.961 (0.935-0.987)	0.940 (0.908-0.973)
Low temperature (P2.5)	11	0.976 (0.948-1.004)	0.943 (0.909-0.979)
Low temperature (P2.5)	12	0.992 (0.959-1.026)	0.946 (0.906-0.989)
Low temperature (P2.5)	13	1.010 (0.968-1.054)	0.950 (0.900-1.004)
Low temperature (P2.5)	14	1.028 (0.975-1.085)	0.955 (0.892-1.022)
High temperature (P97.5)	0	1.008 (0.983-1.034)	1.006 (0.978-1.035)
High temperature (P97.5)	1	1.016 (0.996-1.037)	1.014 (0.991-1.038)
High temperature (P97.5)	2	1.024 (1.007-1.041)	1.022 (1.004-1.041)
High temperature (P97.5)	3	1.032 (1.017-1.046)	1.030 (1.015-1.046)
High temperature (P97.5)	4	1.039 (1.025-1.052)	1.037 (1.023-1.052)
High temperature (P97.5)	5	1.045 (1.031-1.059)	1.044 (1.029-1.059)
High temperature (P97.5)	6	1.051 (1.036-1.066)	1.050 (1.034-1.066)
High temperature (P97.5)	7	1.055 (1.040-1.071)	1.055 (1.038-1.072)
High temperature (P97.5)	8	1.058 (1.043-1.074)	1.058 (1.042-1.075)
High temperature (P97.5)	9	1.061 (1.046-1.076)	1.061 (1.045-1.077)
High temperature (P97.5)	10	1.062 (1.048-1.077)	1.062 (1.047-1.078)
High temperature (P97.5)	11	1.063 (1.048-1.078)	1.063 (1.047-1.080)
High temperature (P97.5)	12	1.063 (1.046-1.081)	1.064 (1.045-1.083)
High temperature (P97.5)	13	1.063 (1.042-1.084)	1.064 (1.041-1.087)
High temperature (P97.5)	14	1.063 (1.037-1.089)	1.063 (1.035-1.092)
Lower humidity (P10)	0	1.017 (1.004-1.030)	1.007 (0.992-1.022)
Lower humidity (P10)	1	1.012 (1.001-1.023)	1.004 (0.991-1.017)
Lower humidity (P10)	2	1.006 (0.997-1.016)	1.002 (0.991-1.013)
Lower humidity (P10)	3	1.001 (0.993-1.009)	1.000 (0.990-1.009)
Lower humidity (P10)	4	0.996 (0.989-1.003)	0.997 (0.989-1.006)
Lower humidity (P10)	5	0.991 (0.985-0.998)	0.996 (0.988-1.003)
Lower humidity (P10)	6	0.988 (0.981-0.994)	0.994 (0.986-1.001)

Exposure contrast	Lag (days)	≤35 months	>35 months
Lower humidity (P10)	7	0.984 (0.977-0.991)	0.992 (0.985-1.000)
Lower humidity (P10)	8	0.981 (0.974-0.989)	0.991 (0.983-1.000)
Lower humidity (P10)	9	0.980 (0.972-0.987)	0.990 (0.982-0.999)
Lower humidity (P10)	10	0.979 (0.971-0.986)	0.990 (0.981-0.999)
Lower humidity (P10)	11	0.979 (0.971-0.986)	0.990 (0.982-0.999)
Lower humidity (P10)	12	0.979 (0.972-0.987)	0.990 (0.982-0.999)
Lower humidity (P10)	13	0.981 (0.974-0.988)	0.991 (0.983-0.999)
Lower humidity (P10)	14	0.983 (0.977-0.990)	0.992 (0.985-1.000)
Lower humidity (P10)	15	0.986 (0.979-0.993)	0.993 (0.986-1.001)
Lower humidity (P10)	16	0.990 (0.982-0.997)	0.995 (0.987-1.003)
Lower humidity (P10)	17	0.993 (0.985-1.001)	0.997 (0.988-1.006)
Lower humidity (P10)	18	0.997 (0.988-1.007)	0.998 (0.988-1.009)
Lower humidity (P10)	19	1.002 (0.991-1.013)	1.000 (0.988-1.013)
Lower humidity (P10)	20	1.006 (0.994-1.019)	1.002 (0.988-1.017)
High humidity (P97.5)	0	0.985 (0.970-1.001)	0.968 (0.950-0.986)
High humidity (P97.5)	1	0.995 (0.981-1.008)	0.977 (0.961-0.993)
High humidity (P97.5)	2	1.004 (0.992-1.016)	0.986 (0.972-1.000)
High humidity (P97.5)	3	1.013 (1.002-1.024)	0.995 (0.983-1.007)
High humidity (P97.5)	4	1.022 (1.012-1.032)	1.003 (0.992-1.015)
High humidity (P97.5)	5	1.030 (1.020-1.039)	1.011 (1.000-1.022)
High humidity (P97.5)	6	1.037 (1.027-1.046)	1.018 (1.007-1.029)
High humidity (P97.5)	7	1.043 (1.033-1.052)	1.024 (1.013-1.035)
High humidity (P97.5)	8	1.047 (1.037-1.057)	1.028 (1.017-1.040)
High humidity (P97.5)	9	1.050 (1.040-1.061)	1.032 (1.020-1.044)
High humidity (P97.5)	10	1.052 (1.041-1.062)	1.033 (1.021-1.045)
High humidity (P97.5)	11	1.052 (1.041-1.062)	1.033 (1.022-1.045)
High humidity (P97.5)	12	1.050 (1.040-1.059)	1.032 (1.020-1.043)
High humidity (P97.5)	13	1.046 (1.037-1.055)	1.029 (1.018-1.039)
High humidity (P97.5)	14	1.041 (1.033-1.050)	1.024 (1.015-1.034)
High humidity (P97.5)	15	1.035 (1.027-1.044)	1.019 (1.010-1.029)
High humidity (P97.5)	16	1.028 (1.020-1.037)	1.013 (1.004-1.022)
High humidity (P97.5)	17	1.021 (1.013-1.030)	1.006 (0.997-1.016)
High humidity (P97.5)	18	1.013 (1.004-1.022)	0.999 (0.988-1.010)
High humidity (P97.5)	19	1.005 (0.994-1.016)	0.992 (0.979-1.004)
High humidity (P97.5)	20	0.996 (0.984-1.009)	0.984 (0.970-0.998)

Values are RR (95% CI). Age groups were defined as 35 months or younger and older than 35 months. No formal interaction test was performed. Low and high temperature correspond to P2.5 and P97.5; lower and high relative humidity correspond to P10 and P97.5. Abbreviations: CI, confidence interval; RR, relative risk.
